# MRI-based deep learning with clinical and imaging features to differentiate medulloblastoma and ependymoma in children

**DOI:** 10.3389/fmolb.2025.1570860

**Published:** 2025-04-28

**Authors:** Yasen Yimit, Parhat Yasin, Yue Hao, Abudouresuli Tuersun, Chencui Huang, Xiaoguang Zou, Ya Qiu, Yunling Wang, Mayidili Nijiati

**Affiliations:** ^1^ Department of Radiology, The First People’s Hospital of Kashi Prefecture, Kashgar, China; ^2^ The Xinjiang Key Laboratory of Artificial Intelligence Assisted Imaging Diagnosis, Varanasi, China; ^3^ The Sixth Affiliated Hospital of Xinjiang Medical University Department of Spine Surgery, Urumqi, China; ^4^ Department of Research Collaboration, R&D Center, Beijing Deepwise and League of PHD Technology Co., Ltd., Beijing, China; ^5^ Clinical Medical Research Center, The First People’s Hospital of Kashi (Kashgar) Prefecture, Kashgar, China; ^6^ First Affiliated Hospital of Xinjiang Medical University Department of Imaging Center, Urumqi, China; ^7^ Department of Radiology, The Fourth Affiliated Hospital of Xinjiang Medical University, Urumqi, China

**Keywords:** deep learning, magnetic resonance imaging, medulloblastoma, ependymoma, T2-weighted imaging

## Abstract

**Background:**

Medulloblastoma (MB) and ependymoma (EM) in children share similarities in terms of age group, tumor location, and clinical presentation, which makes it challenging to clinically diagnose and distinguish them.

**Purpose:**

The present study aims to explore the effectiveness of T2-weighted magnetic resonance imaging (MRI)-based deep learning (DL) combined with clinical imaging features for differentiating MB from EM.

**Methods:**

Axial T2-weighted MRI sequences obtained from 201 patients across three study centers were used for model training and testing. The regions of interest were manually delineated by an experienced neuroradiologist with supervision by a senior radiologist. We developed a DL classifier using a pretrained AlexNet architecture that was fine-tuned on our dataset. To mitigate class imbalance, we implemented data augmentation and employed K-fold cross-validation to enhance model generalizability. For patient classification, we used two voting strategies: hard voting strategy in which the majority prediction was selected from individual image slices; soft voting strategy in which the prediction scores were averaged across slices with a threshold of 0.5. Additionally, a multimodality fusion model was constructed by integrating the DL classifier with clinical and imaging features. The model performance was assessed using a 7:3 random split of the dataset for training and validation, respectively. The key metrics like sensitivity, specificity, positive predictive value, negative predictive value, F1 score, area under the receiver operating characteristic curve (AUC), and accuracy were calculated, and statistical comparisons were performed using the DeLong test. Thereafter, MB was classified as positive, while EM was classified as negative.

**Results:**

The DL model with the hard voting strategy achieved AUC values of 0.712 (95% confidence interval (CI): 0.625–0.797) on the training set and 0.689 (95% CI: 0.554–0.826) on the test set. In contrast, the multimodality fusion model demonstrated superior performance with AUC values of 0.987 (95% CI: 0.974–0.996) on the training set and 0.889 (95% CI: 0.803–0.949) on the test set. The DeLong test indicated a statistically significant improvement in AUC values for the fusion model compared to the DL model (*p* < 0.001), highlighting its enhanced discriminative ability.

**Conclusion:**

T2-weighted MRI-based DL combined with multimodal clinical and imaging features can be used to effectively differentiate MB from EM in children. Thus, the structure of the decision tree in the decision tree classifier is expected to greatly assist clinicians in daily practice.

## 1 Introduction

Primary tumors of the central nervous system (CNS) are very rare in children and have an estimated globally standardized incidence rate of 12 cases per million as of 2018. Despite their rarity, brain tumors are the second leading cause of death after acute lymphoblastic leukemia in children under the age of 15 years (Patel et al., 2018). The estimated globally standardized mortality rate for primary CNS tumors was 0.7 deaths per million in 2018 (Girardi et al., 2019). The majority of pediatric posterior fossa tumors (PPFTs) account for approximately 55%–70% of these cases, where medulloblastoma (MB) and ependymoma (EM) comprise significant portions (Mengide et al., 2023). Both EM and MB are commonly found in the fourth ventricle as well as share similarities in terms of location and morphological appearance on magnetic resonance imaging (MRI) scans, along with heterogeneous enhancement of the solid portion. Differentiating MB from EM in pediatric patients is of paramount importance owing to their distinct biological behaviors, similar clinical presentations, different treatment strategies, and different prognostic implications. MBs are highly malignant embryonal tumors that typically originate in the midline cerebellum and frequently disseminate via the cerebrospinal fluid (CSF). In contrast, EMs originate from the ependymal cells lining the ventricular system, most commonly in the fourth ventricle, and are less prone to CSF dissemination. Clinically, both these types of tumors manifest with signs of increased intracranial pressure. Therapeutically, MBs require maximal resection, craniospinal irradiation, and chemotherapy, with the treatment tailored to molecular risk stratification, whereas EMs require maximal resection followed by focal radiotherapy as craniospinal irradiation is generally unnecessary. The prognoses for these two conditions vary widely, where the MB outcomes depend on molecular subgroups while EM outcomes hinge on resection completeness and tumor location. Accurate differentiation between these types is needed for precise and risk-adapted treatment strategies (de Bont et al., 2008; Menyhárt and Győrffy, 2020).

Currently, the gold standard for tumor classification remains pathological analysis following biopsy or surgical resection (Khatua et al., 2018). However, this method has limitations, such as sampling errors, variations in interpretation, and potential risks of morbidity and mortality associated with biopsies (Almenawer et al., 2015). Hence, preoperative imaging is used for diagnosing, differentiating between the two conditions, and determining the precise anatomical locations of the tumors.

MRI plays a crucial role in the diagnosis of brain tumors owing to its ability to provide detailed images of the brain with high contrast and resolution (Abd-Ellah et al., 2019). MRI allows visualization of the size, location, and characteristics of a brain tumor, thereby aiding clinicians in determining the most appropriate treatment plan for the patient. In particular, T2-weighted imaging (T2WI) is a valuable sequence in MRI scans that is sensitive to the presence of edema, inflammation, and necrosis within the brain tissue (Obenaus and Badaut, 2022). In the diagnosis of brain tumors, T2WI is essential for identifying areas of high signal intensity as they indicate the presence of tumor-associated edema and changes in tissue composition. Additionally, T2WI can help differentiate between different types of brain tumors based on their unique imaging characteristics (Martín-Noguerol et al., 2021).

Radiomics is an advanced computer-aided diagnostic method that utilizes quantitative features extracted from medical images for disease diagnosis, prognosis, and prediction (Lambin et al., 2012). This technique has shown promise in distinguishing between benign and malignant tumors (Zheng et al., 2021), outperforming visual assessments by experienced radiologists. However, the complexity of radiomics analysis, which involves feature extraction, selection, and modeling, can lead to variability in results among different studies. Additionally, existing studies often focus on linear or simplistic algorithms for feature selection, overlooking the intricate non-linear relationships between features across different imaging modalities (Yimit et al., 2023). Further investigations are thus needed to explore these complex interactions while improving the consistency and reliability of radiomics-based diagnostic approaches in clinical practice.

Deep learning (DL) offers a transformative approach by integrating feature extraction, selection, and model development into a unified framework, thereby streamlining radiomics analysis (Xie et al., 2022). DL models have shown exceptional performances in various diagnostic tasks, including distinguishing between benign and malignant renal tumors (Afshar et al., 2019), grading of non-small cell lung cancer (Zhou et al., 2019), and predicting lymph node metastasis in breast cancer (Hosny et al., 2018). Specifically, DL can be used to analyze high-dimensional data and uncover intricate patterns that may be invisible to traditional methods, thus enhancing the diagnostic accuracy and efficiency significantly.

Despite these achievements, there is a notable gap in research regarding the application of DL models to differentiate between MB and EM brain tumors, especially in children. This is an area where the capabilities of DL are yet to be explored and evaluated fully in medical image analyses.

The aim of the present study was to assess the effectiveness of the multimodality fusion model with T2WI-based DL signatures combined with clinical and imaging features in discriminating between MB and EM accurately to provide a useful tool for clinicians and radiologists.

## 2 Methods

The study was conducted at three centers, namely, The First People’s Hospital of Kashi Prefecture, The Third Affiliated Hospital of Xinjiang Medical University, and The First Affiliated Hospital of Xinjiang Medical University, and received ethical approval from their respective institutional review boards. Written informed consent from the participants was waived as this is a retrospective study.

### 2.1 Patient population

A total of 201 patients were enrolled in this multicenter study that encompassed data from three different hospitals:

**Center 1:** The First People’s Hospital of Kashi Prefecture contributed to the majority of the dataset; accordingly, information from 151 patients (93 MB, 58 EM) was collected between 1 December 2009 and 30 July 2024. This dataset formed the backbone of the model training process.

**Center 2:** The Third Affiliated Hospital of Xinjiang Medical University provided information from 37 patients (34 MB, 3 EM) between January 2018 and June 2024.

**Center 3:** The First Affiliated Hospital of Xinjiang Medical University contributed information from 13 patients (all diagnosed with MB) between February 2021 and February 2024.

Given this distribution, it is important to acknowledge that the performance of the proposed model may be influenced by the unequal representation of EM and MB cases. This imbalance could particularly affect the evaluation metrics, such as sensitivity and specificity, potentially leading to a bias in favor of the more prevalent class (MB).

The inclusion criteria for the study were as follows: (1) tumors located in the posterior fossa; (2) histologically verified MB or EM; (3) patients aged under 18 years; (4) availability of axial T2WI sequences; (5) absence of prior brain tumors; (6) availability of relevant clinical information.

The exclusion criteria of the study were as follows: (1) lack of surgical pathology; (2) unavailability of non-enhanced T2WI sequences; (3) poor image quality; (4) previous treatment history.

### 2.2 Imaging data acquisition

The study utilized three 3.0-T MRI scanners. Specifically, Center 1 utilized the Signa Hdx MR scanner from General Electric (USA). The imaging protocol included axial contrast-enhanced T1-weighted imaging (CE-T1WI), axial T2WI, axial fluid-attenuated inversion recovery (FLAIR), sagittal T2WI, as well as contrast-enhanced axial, sagittal, and coronal T1WI sequences. For the axial T1WI sequence, the key parameters were set as follows: repetition time (TR) = 200 ms; echo time (TE) = 12 ms; and slice thickness = 6 mm. The T2WI image acquisition parameters included TR = 3,900 ms; TE = 120 ms; slice thickness = 6 mm; and a field of view (FOV) of 256 × 256 matrices. Centers 2 and 3 utilized the Siemens Trio 3-T scanner from Siemens Healthcare (Erlangen, Germany), with the axial T1WI parameters set at TR = 450 ms; TE = 15 ms; and slice thickness = 5 mm; the T2WI sequence parameters were TR = 5,800 ms; TE = 110 ms; and slice thickness = 5 mm.

Image retrieval from the picture archiving and communication system (PACS) utilized the Digital Imaging and Communications in Medicine (DICOM) format.

### 2.3 Workflow

**FIGURE 1:**
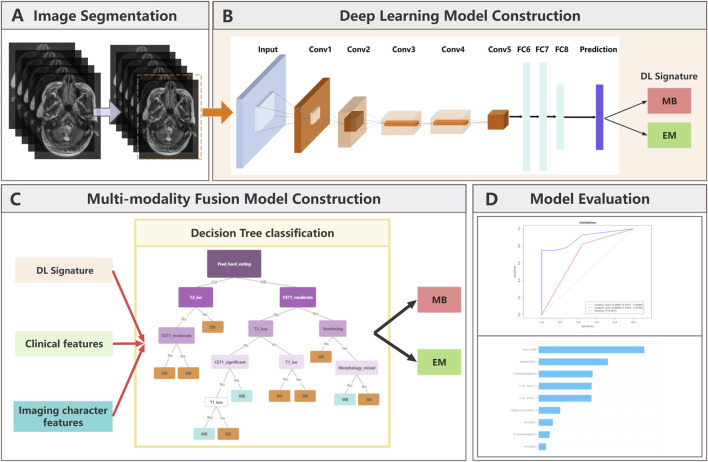
Analytical workflow suggested in this study. Conv, convolutional layer; FC, fully connected layer. **(A)**: Image segmentation workflow; **(B)**: Schematic of the deep learning model architecture; **(C)**: Multi-modality fusion model visualization; **(D)**: Model evaluation dashboard.

The overall workflow of this study is shown in Figure 1. The T2 MRI scans of 190 patients were randomly divided into the training (133 patients) and validation (57 patients) cohorts in the ratio of 7:3. The workflow consisted of region of interest (ROI) segmentation and DL model construction to generate the MB and EM DL classifier, followed by development of the multimodality fusion model to combine the DL signatures, clinical features, and imaging character features using the decision tree method. The DL signatures were the classification results generated from the DL model.

### 2.4 ROI segmentation

The ROIs used for model construction were manually segmented from the MRI scans by a radiologist with 5 years of radiological reading experience and blinded to the histopathological results using the Deepwise Multimodal Research Platform version 2.3 (https://keyan.deepwise.com, Beijing Deepwise and League of PHD Technology Co., Ltd., Beijing, China). A senior radiologist also checked and revised these segmented results.

### 2.5 DL model

The workflow for obtaining the DL signatures is shown in Figure 1. The ROIs of the 2D slices with tumors were used as input data per patient to the DL model to fully utilize the information from all layers.

To enhance the generalization capability and classification performance of the model, we standardized the imaging data from the different scanners; this included normalizing pixel values to the range of [0, 255] and adjusting the resolution to [1, 1, 1]. We adopted the pretrained AlexNet architecture comprising five convolutional layers and three fully connected layers to fine-tune the model using the training cohort. Data augmentation techniques, such as mirroring, elastic deformation, rotations, scaling, resampling, brightness and contrast adjustments, gamma correction, Gaussian noise addition, and center cropping, were applied to the images prior to training to enrich the dataset.

The network was trained with the cross-entropy loss function and Adam optimizer with a learning rate of 0.01 and implemented on the Deepwise Multimodal Research Platform version 2.3 (https://keyan.deepwise.com, Beijing Deepwise and League of PHD Technology Co., Ltd., Beijing, China). As depicted in Figure 1, the DL signatures needed to build the models for differentiating MB and EM were obtained as follows. The DL model was first trained on the 2D slices of all patients in the training group to effectively increase the sample size; in addition, K-fold cross-validation was incorporated in the DL classifier training set to ensure that there was no overlap between the training and test sets for promoting generalizability. The prediction results were first acquired by implementing the DL model on all 2D slices; then, the classification result of each patient was obtained by ensembling the prediction results on all the slices of the patient.

To address the potential impact of class imbalance during model training, we employed several strategies. First, data augmentation techniques were applied to the training cohort, which aimed to enhance the representation of the less-frequent class (EM) and improve the model’s ability to learn distinguishing features from both classes more effectively.

Additionally, we implemented K-fold cross-validation for more robust evaluation of the model’s performance across various subsets of the data, thereby helping to mitigate the effects of class imbalance on the overall evaluation metrics.

**FIGURE 2:**
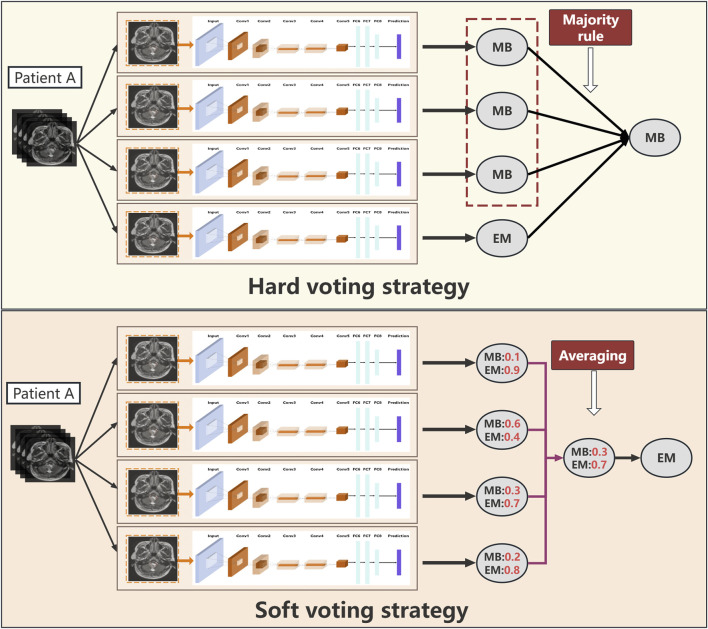
Workflows of the hard and soft voting strategies.

As depicted in Figure 2, we experimented with two voting strategies: the hard and soft voting strategies. For the soft voting strategy, we considered the prediction scores of the images based on the DL model (ranging from 0 to 1) as inputs to calculate their average value as the final prediction score for each patient; here, a threshold of 0.5 was used for classification. For the hard voting strategy, we followed the majority rule; here, for each patient, the classifier prediction for each image slice was considered as one vote, and the prediction that accounted for over half of all votes was considered as the final result for the patient.

### 2.6 Multimodality fusion model

In this step, we first collected the clinical characteristics of each patient, including gender, body mass index (BMI), age, and symptoms like headache, vomiting, and gait instability. A radiologist then conducted a detailed evaluation of the MRI scans and assessed key imaging features, such as the involved quadrants, orbital area involvement, lesion shapes and borders, and signal intensity, from the T1WI and T2WI. Then, a senior neuroradiologist evaluated these assessments for confirmation; this evaluation was the basis for recording the imaging characteristics relevant to routine diagnosis, including lesion morphology, shape regularity, signal intensities on the T1WI and T2WI, enhancement patterns on the contrast-enhanced T1WI (CET1), and apparent diffusion coefficient (ADC) values.

Next, we implemented the multimodality fusion model to validate the diagnostic ability to differentiate MB from EM by combining the DL signatures, clinical characteristics, and imaging features. The seven clinical features, six imaging features, and DL signatures were first combined and selected using the least absolute shrinkage and selection operator (LASSO) feature selection algorithm with parameter C = 0.05; it is worth noting that K-fold cross-validation was also incorporated using the LASSO algorithm. After applying the feature selection, the selected features were combined with the DL model prediction results using the decision tree model. This model was trained by considering the Gini loss function.

### 2.7 Statistical analysis and model evaluation

#### 2.7.1 Statistical analysis

The performances of the DL classifier and multimodality fusion model were evaluated using statistical metrics, including area under the curve (AUC), accuracy (ACC), sensitivity (SEN), specificity (SPE), positive predictive value (PPV), and negative predictive value (NPV). The confidence intervals (CIs) for the AUCs were calculated using the DeLong method to assess the robustness and reliability of the model. For continuous variables, the Mann–Whitney U test was applied, while the categorical variables were analyzed using the Chi-squared or Fisher’s exact test. A *p*-value <0.05 was considered to be statistically significant for all analyses. Finally, MB was designated as the positive class and EM was considered as the negative class.

#### 2.7.2 Model evaluation

The models were trained and validated using a 7:3 random split of the data. In addition to this split, we employed K-fold cross-validation while training the DL classifier; this ensures that the model is assessed on multiple subsets of the data, thereby enhancing its generalizability to unseen cases. Each fold allows a robust evaluation of the model’s performance, thereby providing a more reliable estimate of its predictive capabilities.

To rigorously compare the receiver operating characteristic (ROC) curves of different models, the DeLong test was employed; this method provides a statistically robust comparison of the AUCs between two correlated ROC curves, enabling the evaluation of whether observed differences in the model performance were statistically significant. The DeLong test results were expressed as *p*-values, which served as critical indicators of the likelihood of observing the current or a more extreme difference under the null hypothesis that the two models have equivalent diagnostic accuracies.

For the hard and soft voting strategies, the performance metrics were compared on both the training and validation sets. The hard voting strategy demonstrated superior diagnostic accuracy than the soft voting approach, as reflected by higher AUC values with corresponding confidence intervals. The multimodality fusion model combining DL signatures, clinical features, and imaging features using the decision tree framework was further evaluated for statistical significance to confirm its enhanced diagnostic performance over the standalone DL classifier.

## 3 Results

### 3.1 Interpretation of the clinical imaging features

As is shown in Table 1, a total of 201 patients were included in this study. Among these, male patients were more common than female patients (*p* = 0.017). The BMI, vomiting, headache, and gait instability features did not show any statistical differences. In terms of morphology, solid components were common in MB, whereas cystic or mixed masses were more common in EM (*p* = 0.005). On the contrast-enhanced T1WI sequences, MB showed a predominantly significant enhancement pattern, whereas EM mainly showed mild or moderate enhancement (*p* < 0.001). The ADC value for MB was significantly lower than that for EM (*p* < 0.001). The other features like shape and signal intensities on the T1WI and T2WI showed no statistical differences.

**TABLE 1:** Clinical and imaging characteristics used in this study.

Variable	MB (n = 136) *M* (*P*_25_, *P*_75_), n (%)	EM (n = 65) *M* (*P*_25_, *P*_75_), n (%)	*H*/*χ* ^2^	*p*
Gender			5.745	0.017
Male	81 (59.6)	27 (41.5)		
Female	55 (40.4)	38 (58.5)		
Body mass index	21.75 (19.40, 23.00)	21.50 (19.40, 23.00)	−0.385	0.700
Age (years)	5.00 (4.00, 7.00)	5.00 (3.00, 7.00)	−1.722	0.085
Headache			2.944	0.086
No	50 (36.8)	16 (24.6)		
Yes	86 (63.2)	49 (75.4)		
Vomiting			0.019	0.889
No	76 (55.9)	37 (56.9)		
Yes	60 (44.1)	28 (43.1)		
Gait instability			3.375	0.066
No	69 (50.7)	24 (36.9)		
Yes	67 (49.3)	41 (63.1)		
Morphology			10.453	0.005
Solid	79 (58.1)	22 (33.8)		
Cystic	26 (19.1)	21 (32.3)		
Mixed	31 (22.8)	22 (33.8)		
Shape			1.003	0.309
Irregular	67 (49.3)	37 (56.9)		
Regular	69 (50.7)	28 (43.1)		
T1			0.464	0.793
Low	60 (44.1)	29 (44.6)		
Iso	39 (28.7)	16 (24.6)		
High	37 (27.2)	20 (30.8)		
T2			3.421	0.181
Low	32 (23.5)	19 (29.2)		
Iso	49 (36.0)	15 (23.1)		
High	55 (40.4)	31 (47.7)		
Contrast-enhanced T1			36.393	<0.001
Mild	30 (22.1)	18 (27.7)		
Moderate	26 (19.1)	36 (55.4)		
Significant	80 (58.8)	11 (16.9)		
Apparent diffusion coefficient	0.50 (0.41, 0.59)	0.87 (0.83, 0.94)	11.059	<0.001

### 3.2 Evaluation of predictive voting strategies for different DL models

For the DL model, we adopted two voting strategies for predicting the results of the slice assembly: hard and soft voting. We assessed those two voting strategies using the AUC and confusion matrix. As shown in Table 2, the DL results generated by hard voting were superior to those obtained using soft voting across all model performance evaluation criteria, except specificity, on both the training and test sets. The model based on hard voting achieved AUC values of 0.712 (95% CI: 0.625–0.797) on the training set and 0.689 (95% CI: 0.554–0.826) on the test set; in comparison, the model based on soft voting achieved AUC values of 0.705 (95% CI: 0.615–0.794) on the training set and 0.633 (95% CI: 0.497–0.767) on the test set. Moreover, the DL prediction performances based on hard voting on the training and test sets showcase remarkable model generalization. Based on the model performances, we chose the prediction results generated by the DL model based on hard voting as the inputs to the multimodality fusion model.

**TABLE 2:** Diagnostic performances of the image-based deep learning models with the hard and soft voting strategies.

Voting strategy	Dataset	AUC (95% CI)	ACC	SEN	SPE	NPV	PPV
Hard voting	Training set	0.712 (0.625–0.797)	0.786	0.936	0.487	0.792	0.785
	Test set	0.689 (0.554–0.826)	0.717	0.821	0.5556	0.6667	0.742
Soft voting	Training set	0.705 (0.615–0.794)	0.778	0.923	0.487	0.760	0.783
	Test set	0.633 (0.497–0.767)	0.674	0.821	0.444	0.615	0.697

ACC, accuracy; AUC, area under the curve; CI, confidence interval; NPV, negative predictive value; PPV, positive predictive value; SEN, sensitivity; SPE, specificity.

### 3.3 Performance differences between the image-based DL and multimodality fusion models

**FIGURE 3:**
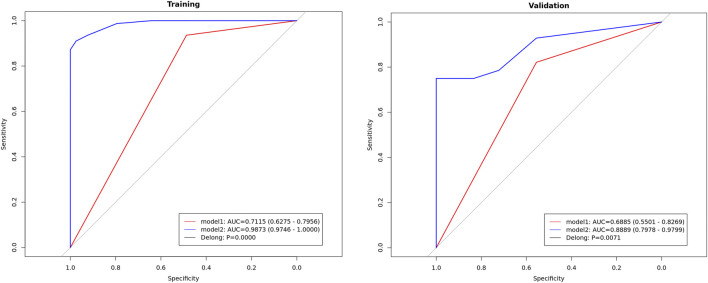
ROC curves of the image-based deep learning model and multimodality fusion model.

For the multimodality fusion model, we selected five imaging features (signal intensities on the T1WI and T2WI, morphology, shape regularity, and enhancement pattern on CET1), one clinical characteristic (vomiting), and one DL signature (Pred_hard_voting). The diagnostic performances of the multimodality fusion model on the training and test sets are shown in Table 3. The AUC values for this model were 0.987 on the training set and 0.889 on the test set, presenting considerable model generalization. The ROC curves were also compared between the DL model based solely on images and multimodality fusion model shown in Figure 3, for which the performance of the multimodality fusion model was noted to surpass that of the image-based DL model, indicating a better discrimination ability. Furthermore, the multimodality model showed notable capability to designate positive and negative individuals based on the sensitivity of 0.910 and specificity of 0.974 on the training set.

**TABLE 3:** Diagnostic performance of the multimodality fusion model.

Model	Dataset	AUC (95% CI)	ACC	SEN	SPE	NPV	PPV
Multimodality fusion model	Training set	0.987 (0.975–1.000)	0.932	0.910	0.974	0.844	0.986
	Test set	0.889 (0.798–0.980)	0.848	0.750	1.000	0.720	1.000

ACC, accuracy; AUC, area under the curve; CI, confidence interval; NPV, negative predictive value; PPV, positive predictive value; SEN, sensitivity; SPE, specificity.

### 3.4 Decision tree structure and feature importance of the multimodality fusion model

**FIGURE 4:**
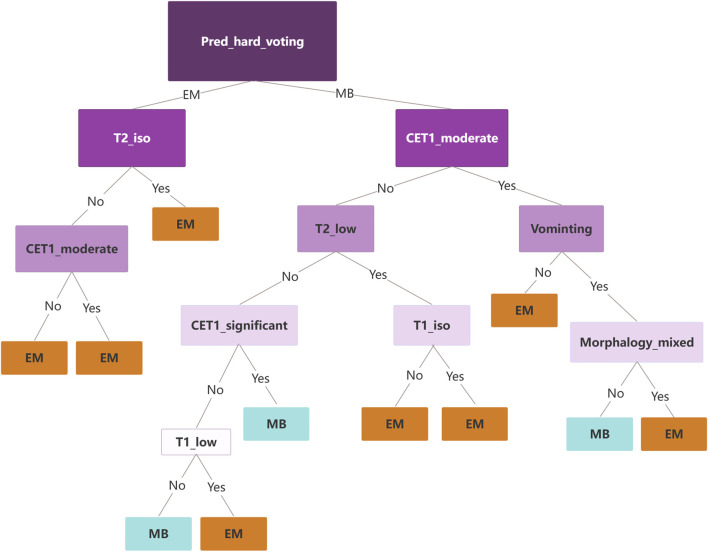
Visual decision tree structure of the multimodality fusion model.

**FIGURE 5:**
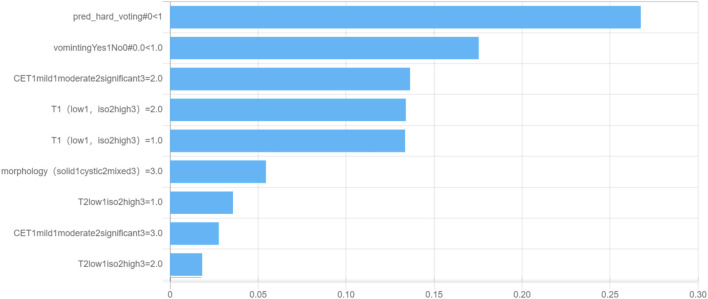
Feature importance for the multimodality fusion model.

To understand the relative influences of the model variables, we visualized the decision tree structure in the decision tree classifier (Figure 4). The distributions of the features categorized by the dependent variable “label” are presented in Figure 5. Notably, the DL signature was identified as the most relevant predictive factor, following which the feature indication of “vomiting,” CET1, and signal intensity on the T1WI contributed significantly to the Gini information increment.

## 4 Discussion and limitations

To distinguish between MB and EM, we developed a DL model integrating the radiomics features of T2WI with clinical and imaging characteristics. The model shows promising performance, with a hard voting strategy outperforming the soft voting approach to yield AUC values of 0.71 on the training set and 0.69 on the test set. Furthermore, the multimodality fusion model achieved impressive AUC values of 0.99 on the training set and 0.89 on the test set. These results highlight the potential of combining radiomics with clinical data in alignment with previous studies that emphasize the importance of multimodal approaches in improving the diagnostic accuracies of brain tumors. The superior results from the multimodality fusion model compared to the image-based DL model suggest that integrating diverse data sources can better capture complex tumor characteristics, offering a promising strategy for clinical decision-making.

MB and EM are prevalent pediatric brain tumors with notable similarities in their imaging and clinical presentations. However, MB and EM differ in their treatment approaches and prognoses. MB treatment typically involves surgical resection, radiation therapy, and adjunctive chemotherapy post-surgery along with radiation (Sun et al., 2020). In contrast, EM exhibits limited sensitivity to chemotherapy, primarily relying on surgery and radiation for treatment (Gwynne et al., 2022). Prognostically speaking, MB is associated with poor outcomes and high metastatic risk with reported 5-year survival rates of 30%–50% (Rutkowski et al., 2005). Conversely, EM generally has a more favorable prognosis.

Accurate preinterventional diagnosis is essential for optimal tumor management. Although surgical pathological biopsy is commonly used for accurate diagnosis, it entails inherent risks such as hemorrhage and damage to normal brain tissue. Hence, the importance of non-invasive diagnostic approaches cannot be overstated in clinical practice. Radiomics leverages machine-generated quantitative features from medical images for disease evaluation and shows potential in diverse medical areas. However, traditional radiomics analysis, with its complex steps like manual feature extraction and selection, often yields inconsistent results owing to stochasticity (Lambin et al., 2012). In contrast, DL directly learns relevant information from images by combining feature extraction, selection, and model building within a single neural network via end-to-end learning (Gojo et al., 2020). In our previous study (Yimit et al., 2024), we explored the potential of radiomics for differentiating MB from EM and achieved considerable distinguishability; however, there were problems with delineating lesions as well as extracting and selecting features, which limited its clinical application. Furthermore, our previous study does not involve the use of imaging and clinical features in decision-making. The proposed integrated approach streamlines radiomics analysis, offering a more efficient and effective method for deriving insights from medical imaging data (Rudà et al., 2022).

The present study is a pioneering effort to utilize DL with clinical and imaging features to distinguish MB from EM, thereby streamlining radiomics analysis by extracting features directly from MRI scans via end-to-end learning. In this study, different imaging features, clinical features, and T2WI-extracted features were incorporated to establish a multimodal fusion model for classification. Among the clinical features, gender showed a slight statistical difference, with male patients being more common than female patients, consistent with previous studies (Weil et al., 2017). There were no statistically significant differences in terms of age, BMI, headache, vomiting, and gait instability. Regarding the imaging features, MB exhibited a higher prevalence of solid masses, while cystic and mixed masses were less common; in contrast, EM showed a higher prevalence of cystic and mixed masses, with solid masses being less frequent; this difference was statistically significant (*p* = 0.041). The predominance of solid masses in MB can be attributed to its cellular composition characterized by densely packed cells (Zhou et al., 2023), while the histological features of EM, such as necrosis and cyst formation, contribute to the prevalence of cystic and mixed masses in these tumors (Zhao et al., 2023).

In the contrast-enhanced T1WI sequences, EM predominantly showed moderate and mild enhancement, whereas MB had less-common mild and moderate enhancement patterns. Significant enhancement was more prevalent in MB with a statistically significant difference (*p* < 0.001). This distinct contrast-enhancement pattern can be attributed to the histological characteristics of these tumors. MB is known for its high vascularity and rapid growth, so it typically exhibits significant enhancement owing to pronounced uptake of the contrast agent (Xia et al., 2024). Conversely, the more heterogeneous nature of EM may contribute to the observed moderate and mild enhancement patterns in this tumor type. In addition, if CE-T1WI were used instead of T2WI in the DL model, it is likely that the results could be improved, potentially enhancing the model’s predictive power and specificity. Thus, the diagnostic efficiency of CE-T1WI should be investigated in future research.

The ADC value is significantly lower in MB than EM, with a *p*-value of less than 0.001. These differences can be attributed to variations in the cellular density and microscopic structure of these tumors. MB tumors are characterized by higher cellularity and densely packed cells, which restrict the diffusion of water molecules and result in lower ADC values. Conversely, EM tumors typically have lower cellularity and more open cellular structures, leading to higher ADC values. This result is consistent with the findings of previous studies (Medulloblastoma, 2019).

In the present study, predictive voting strategies like hard and soft voting were used, which are commonly employed in ensemble learning, particularly in the context of DL models. In hard voting, each base model or classifier in an ensemble makes a prediction based on the input data; the final prediction is then determined by a majority vote, where the class that receives the most “votes” from the individual classifiers is selected as the predicted class label (Weil et al., 1998; Gilbertson and Ellison, 2008). On the other hand, soft voting involves considering the predicted probabilities or confidence scores provided by each classifier individually for the various classes. Rather than selecting the majority vote, soft voting averages these probabilities to arrive at a final prediction; then, the class with the highest average probability across all classifiers is chosen as the predicted class label (Koeller and Rushing, 2003; Waldron and Tihan, 2003).

The decision tree classifier is a popular machine learning algorithm that utilizes a tree-like model of decision rules to make predictions. The Gini index shows that DL is the most important predictive factor in our study, followed by the feature indicators vomiting, CET1, and T1 enhancement pattern. In clinical practice, doctors can use these indicators and logic to accurately diagnose MB and EM.

The observation that “vomiting” contributes significantly to the Gini information increment in the decision tree model, despite its lack of statistical significance in traditional analyses (Table 1), reveals a critical methodological distinction. This reflects the different principles underlying statistical tests and machine learning-based feature-importance assessments.

Decision tree models prioritize features that reduce impurity, such as the Gini index, thereby capturing complex non-linear interactions and conditional dependencies among the variables. Although “vomiting” as a feature may lack a robust independent relationship with the outcome variable, its integration with other features (e.g., CET1 intensity or T1WI signal) enhances the model’s predictive capacity. This divergence arises because traditional significance testing typically evaluates features in isolation under linear assumptions, whereas decision trees uncover synergistic effects and latent relationships that remain undetected in parametric models.

The contextual relevance of “vomiting” suggests its role as a secondary or interaction-based predictor, contributing to unique decision pathways within the model. Its inclusion in our model demonstrates the utility of decision trees in exploring multidimensional non-linear data relationships.

This observation underscores the importance of complementing traditional statistical approaches with machine learning methods. By integrating both paradigms, researchers can achieve a more nuanced understanding of feature relevance, fostering advancements in multimodality modeling and data interpretability.

In the results, the low specificity of the DL model refers to its tendency to make broad and generalized predictions that may lack precision for certain tasks. Although DL models excel in recognizing patterns across large datasets, they often struggle with fine-tuning of predictions for specific nuanced cases. This is partly attributable to their reliance on vast amounts of training data, which can cause overfitting to common patterns while underperforming on rare or edge cases. Enhancing the specificity requires tailored model architectures, improved diversity of the training data, and better regularization techniques to focus on more precise decision boundaries.

Although our study demonstrates promising discrimination capabilities using DL in combination with clinical and imaging features, several limitations should be noted. Despite collecting patient data from three centers, the sample size remained relatively small, without a separate external validation set. Future research should thus focus on large-scale multicenter studies to validate these findings. Additionally, the absence of pathological whole-slide images in our study suggests that incorporating such data could enhance the model’s differentiation performance. Overfitting remains a challenge with most learning-based models and necessitates further investigation, particularly through multicenter studies with balanced sample sizes, to address this issue.

## 5 Conclusion

The T2WI-based DL model combining clinical and imaging features is shown to be able to differentiate MB from EM in children in this study. The proposed model exhibits high predictive accuracy and stability, showcasing its potential to improve precision in oncology while offering practical applications in clinical settings. In addition, the use of CE-T1WI instead of T2WI in the DL model is expected to provide improved results, which is suggested as the direction for further investigations.

## Data Availability

The original contributions presented in the study are included in the article/Supplementary Material, and further inquiries can be directed to the corresponding authors.
